# The management of mirror foot polydactyly: A case report

**DOI:** 10.1016/j.ijscr.2022.106780

**Published:** 2022-01-17

**Authors:** Panji Sananta, Ficky Fajar Sahdiniar, Lasa Dhakka Siahaan

**Affiliations:** a Teaching Staff of Orthopaedic and Traumatology Department, Faculty of Medicine Universitas Brawijaya, RSUD Dr. Saiful Anwar, Jl. Jaksa Agung Suprapto 2, Malang, 65111, East Java, Indonesia; b Resident of Orthopaedic and Traumatology Department, Faculty of Medicine Universitas Brawijaya, RSUD Dr. Saiful Anwar, Jl. Jaksa Agung Suprapto 2, Malang, 65111, East Java, Indonesia; c Orthopaedic and Traumatology Department, Faculty of Medicine Universitas Brawijaya, RSUD Dr. Saiful Anwar, Jl. Jaksa Agung Suprapto 2, Malang, 65111, East Java, Indonesia

**Keywords:** Mirror foot, Preaxial polydactyly, Treatment, Congenital anomaly, Children

## Abstract

**Background:**

Mirror foot or mirror image duplication of the foot is an extreme form and very rare congenital anomaly. There are limited management recommendations, and most cases are treated before walking age. We present the clinical findings, surgical treatment, and results of a rare case of mirror foot polydactyly.

**Case presentation:**

A five-month-old girl with bilateral mirror foot was referred to our orthopaedic department. She was born full-term by the caesarian section and there was no family history of similar skeletal abnormalities and no history of drug or radiation exposure during gestation. The child had eight toes on the right foot and seven toes on the left with fully developed metatarsal, proximal, middle, and distal phalanges. Radiographs confirmed the diagnosis of mirror foot with a full complement of normal lateral toes and three additional complete rays medial to the right foot and two additional complete rays medial to the left foot. The patient underwent ray resection and concurrent reconstruction of the medial arch of the foot. A medial longitudinal incision was used to excised the right medial three rays and left medial two rays. The target of this surgical intervention was for aesthetic or cosmetic reasons and enabling the patient to allow shoe wear.

**Conclusion:**

Mirror-foot abnormalities are distinctly uncommon entities and represent extreme forms of congenital duplication of the preaxial polydactyly spectrum. Treatment on age of five-month-old with medial longitudinal incision had a satisfying clinical and radiological results.

## Highlights

• An extreme form of preaxial foot polydactyly has been coined the term preaxial mirror polydactyly is rare congenital anomaly.
• Few cases have been reported, and there is little consensus concerning the optimal technique for surgical management.
• For the diagnosis of mirror foot, there has to be a mirror image polydactyly on the medial aspect of the foot.

## 1 Introduction

Polydactyly, one of the prevalent congenital foot deformities, is a duplication of a toe in hand or foot that occurs bilaterally in 40–50% of patients. Nearly 80% of these patients have postaxial polydactyly (duplication of the fifth toe), which often is not symmetric. The Dutch Physician Theodor Kerckring first proposed the term polydactyly in 1670 [1].

The overall incidence of polydactyly is 1.7/1000 births, with a significantly higher incidence among the African-American population [2], [3]. Polydactyly can occur as an isolated congenital condition or part of a genetic syndrome with other multiple congenital anomalies. The reported anomalies associated with mirror foot are mirror hands, tibial hemimelia (various degrees of hypoplasia of tibia) [ 4], fibular dimelia (absent tibia with duplication of the fibula), and Laurin–Sandrow syndrome [5].

Temtamy and McKusick have described polydactyly based on the location of the extra digit such as Preaxial (medial ray), central, and postaxial (lateral ray). Postaxial polydactyly occurring in 80% of the patients is often asymmetric. Preaxial polydactyly affects the big toe and occurs in 15% of patients, while central duplication occurs in the remaining 5%, often duplicating a hypoplastic metatarsal ray [6].

An extreme form of preaxial foot polydactyly has been coined the term mirror foot or preaxial mirror polydactyly or has been considered the same as diplopodia, a rare congenital anomaly [7]. Fukazawa and Kawabata stated that 28 cases had been reported in the English literature, among which only seven cases have been documented for their treatment [8]. For the diagnosis of mirror foot, there has to be a mirror image polydactyly on the medial aspect of the foot. However, there is a debate regarding the fulfillment of the criteria, particularly the duplication of all the tarsal and metatarsal bones on the medial side of the foot. There is no universal agreement on what constitutes a mirror foot. While some authors consider mirror foot as any foot with mirror image polydactyly, others believe that mirror duplication of all the skeletal elements of the foot on the tibial aspect must be present to fulfil the criteria for a mirror foot. There are others who suggest that feet with supernumerary rays situated preaxially (i.e., medial to the first ray), but with characteristics of postaxial toes be considered as mirror feet. Preaxial mirror polydactyly is distinctly rare with only 30 cases reported in literature. Treatment consists of excision of the extra rays to allow fitting of shoes [9]. This is often done via racket-type incisions on the border of the extra digits. The most normal-looking digits are usually preserved. In the central type of the mirror foot, the resection of the middle rays will produce a functional and cosmetic foot [10].

We report a case of bilateral preaxial mirror polydactyly with eight metatarsals in the right foot and seven metatarsals in the left foot of a 5-month-old female child. We report this case for its rarity, unusual presentation, and successful surgical treatment. This report has followed SCARE checklist and guidelines, and consent was given by our patient's guardian regarding the data obtained in this case would be submitted for publication [11].

## 2 Case presentation

**Fig. 1:**
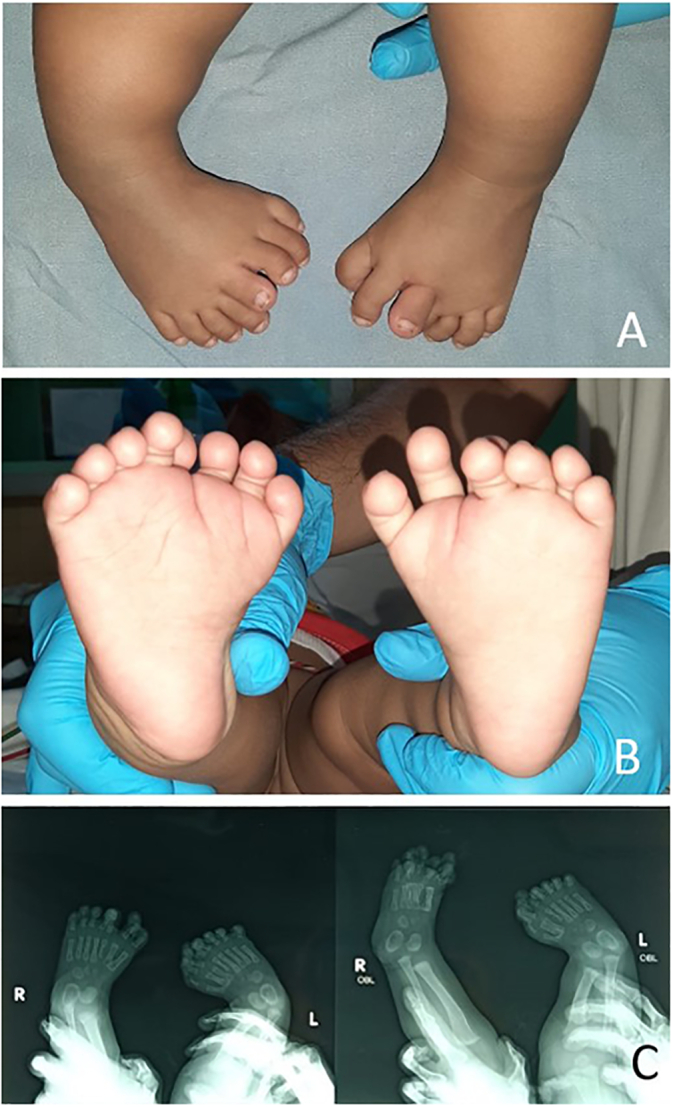
A) Dorsal view of the foot shows eight toes on the right foot and seven toes on the left foot B); Plantar view of the foot; C) Radiological studies shows fully developed metatarsal, proximal, middle, and distal phalanges.

A five-month-old girl with bilateral mirror foot was referred to our orthopaedic department. She was born full-term by the caesarian section and was the firstborn of the couple. There was no family history of similar skeletal abnormalities and no history of drug or radiation exposure during gestation. The clinical and radiological assessment did not reveal any other skeletal abnormalities (Fig. 1).

The child had eight toes on the right foot and seven toes on the left (Fig. 1) with fully developed metatarsal, proximal, middle, and distal phalanges. Radiographs were obtained at the age of five months and confirmed the diagnosis of mirror foot with a full complement of normal lateral toes and three additional complete rays medial to the right foot and two additional complete rays medial to the left foot.

**Fig. 2:**
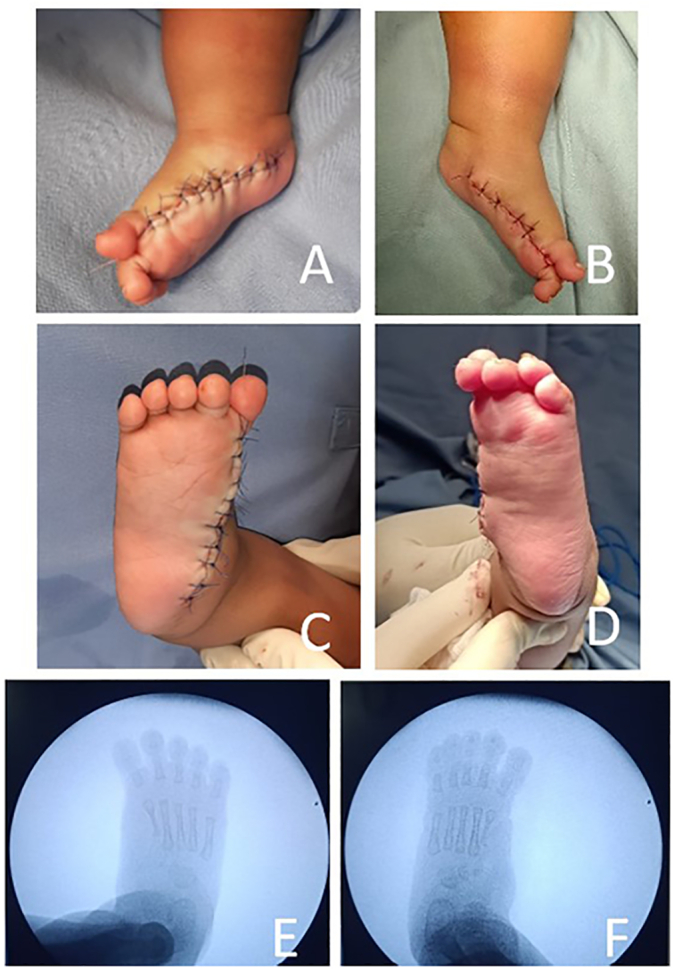
Post operative picture A) Medial view of right foot B) Medial view of left foot; C) Plantar view of right foot; D) Plantar view of right foot; E) Radiological imaging of right foot; F) Radiological imaging of left foot.

Surgery was performed under general anesthesia, and a tourniquet was applied. The patient underwent ray resection and concurrent reconstruction of the medial arch of the foot. We decided to remove the first, second, and third toe from the medial side of the right foot and then the first and second toe from the left foot. A medial longitudinal incision was used to excised the right medial three rays and left medial two rays. The abnormal mid-foot cartilage was refashioned, and the excised accessory tendons were used to reinforce the ligaments of the medial arch (Fig. 2). Duplication of the tibialis anterior tendon was observed and inserted into the medial polydactyly rays. This tendon was relocated to its normal insertion on the medial cuneiform to reinforce arch elevation. The toes were removed up to the metatarsal portion. Furthermore, for the restoration of stability, the intermetatarsal ligaments were sutured.

Post-operative clinical and radiological examinations were subsequently performed to observe the surgery's result. It is difficult to assess the functional capabilities of this patient since the patient is still five-month-old and has not been able to walk yet. The target of this surgical intervention was for aesthetic or cosmetic reasons and enabling the patient to allow shoe wear. Follow-up is required to assess the functional development of this patient later in life.

## 3 Discussion

The definition of mirror foot is diverse due to the variability in its patterns. Also, different terminologies are used to describe this specific type of foot duplication. The true definition of mirror foot is author-dependent, and the literature review shows an important variability in the patterns of mirror polydactyly [12]. While some authors consider mirror foot as any foot with mirror-image polydactyly; others believe that mirror duplication of all the skeletal elements of the foot on the tibial aspect must be present to fulfil the criteria for diagnosing a mirror foot [13], [14].

Mirror-foot abnormalities are distinctly uncommon entities and represent extreme forms of congenital duplication of the preaxial polydactyly spectrum [15]. There are limited surgical management recommendations in the reported literature for this uncommon anomaly with functional and aesthetic implications.

After referring to the classification of the mirror hand by Al-Qattan et al. [16], Fukuzawa et al. [8] classified mirror foot with the classification of mirror hand by changing ulna into fibula and radius into tibia (Table 1). Fibula dimelia was observed only in less than half of the reported cases of mirror foot with the remainder having either a normal or hypoplastic, bowed tibia. Meanwhile almost all mirror hands are associated with ulnar dimelia. Mirror hands have seven or eight digits and no thumbs. Mirror foot, however, is more variable and may have no obvious hallux or a fused central hallux and seven or eight digits [17]. Based on this classification, our patient was categorized to the multiple foot type since she has complete multiplication of the toes up to the metatarsal portion and normal tibia and fibula.

**Table 1:** Classification of mirror foot [8].

Type	Name	Clinical features
1	Fibula dimelia	Multiple toes with two fibulae
		A: each fibula is well formed
		B: the preaxial fibula is hypoplastic
2	Intermediate type	Multiple toes with two fibulae (one of the fibulae is vestigial) and a tibia
3	Intermediate type	Multiple toes with one fibula and a tibia
		A: the tibia is well formed
		B: the tibia is hypoplastic
4	Syndromal mirror feet	Bilateral multiple toes in complex syndactyly
		Mirror hands and nasal defects are also characteristic
		A: Sandrow syndrome
		B: Martin syndrome
5	Multiple foot	Complete duplication of the foot, including the hallux, with a normal leg
6	No tibia and fibula	

Surgical treatment of the mirror foot is less complex than its upper limb equivalent due to differences of function between hand and foot [17]. Essentially, there must be an initial surgical reduction in the number of toe rays, coupled with tendon transfers and ligament reconstruction as necessary to protect the medial arch [18]. Hallux varus, and persistent widening of the forefoot, as is seen in pre-axial polydactyly of the foot, should be anticipated when planning the reconstruction and may require further correction [8]. When the child is older, further procedures may be required to address any tibial discrepancy.

We believed that early surgical corrections in the pre-ambulatory phase to reduce the mirror deformity, together with reconstructive arch support procedures, have produced good functional and aesthetic outcomes. Up to this point, no additional reconstructive surgeries were required, and the follow-up continues within a multidisciplinary team. Physiotherapists and occupational therapists should be involved with early and regular gait assessments to identify, prevent, and improve any abnormalities.

This case report presents a rare case of bilateral preaxial mirror polydactyly and the successful surgical method we did to the patient. However, there are some limitations present in this study. First, this study design is not enough to give a high level of evidence. Further Randomized Controlled Trial or prospective cohort study is necessary to give a higher level of evidence. However, due to the rarity of the case, a prospective analytical study is difficult to be conducted. Second, due to the early age of the patient, the pre and post-operative functional status of the foot is difficult to be assessed. Thus the functional surgery outcome could not be concluded. Lastly, there is no further follow up done to this patient caused by the loss of contact, making the sustainability of the surgery's outcome difficult to conclude.

## 4 Conclusions

Mirror-foot abnormalities are distinctly uncommon entities and represent extreme forms of congenital duplication of the preaxial polydactyly spectrum. Few cases have been reported, and there is little consensus concerning the optimal technique for surgical management. We had satisfying clinical and radiological results after the treatment of a five-month-old with medial longitudinal incision.

## Funding

This research did not receive any specific grant from funding agencies in the public, commercial, or not-for-profit sectors.

## Consent

Written informed consent was obtained from the patient's guardian for publication of this case report and accompanying images. A copy of the written consent is available for review by the Editor-in-Chief of this journal on request.

## Ethical approval

This study has been reviewed and approved by the authors' Institutional Review Board.

## Registration of research studies

This case report is not “First in Man” study.

## Guarantor

Panji Sananta

Orthopaedics and Traumatology Department, Faculty of Medicine, Universitas Brawijaya-RSUD Dr. Saiful Anwar.

Jl. Jaksa Agung Suprapto No.2, Klojen, Malang 65,111, East Java, Indonesia.

E-mail address: panjisananta@ub.ac.id

## CRediT authorship contribution statement

Panji Sananta: conceptualization, writing original draft preparation, supervision, final approval

Ficky Fajar Sahdiniar: data collecting, data interpretation, writing the paper and editing, final approval

Lasa Dhakka Siahaan: data collecting, data interpretation, writing the paper and editing, final approval

## Declaration of competing interest

We declare that they have no known competing financial interests or personal relationships that could have appeared to influence the work reported in this paper.
